# Nutrition and physical activity in cancer patients: a survey on their information sources

**DOI:** 10.1007/s00432-022-04282-w

**Published:** 2022-08-22

**Authors:** Sebastian Josef Boesenecker, V. Mathies, J. Buentzel, J. Huebner

**Affiliations:** 1 Clinic for Internal Medicine II, University Hospital, Bachstraße 18, 07743 Jena, Germany; 2 University Tumor Center, University Hospital, Jena, Germany; 3 Clinic for Otorhinolaryngology, Head Neck Surgery, Suedharz Klinikum, Nordhausen, Germany

**Keywords:** Oncology, Access to information, Healthy lifestyle, Physical activity, Nutrition

## Abstract

**Background:**

Many cancer patients suffer from problems concerning nutrition and physical activity (PA) during and after their treatment. Forwarding reliable health information could help to alleviate severe symptoms. The present study aimed to examine cancer patients’ commonly used information sources on nutrition and PA.

**Methods:**

An anonymous questionnaire was developed and distributed to German cancer patients in different settings. In total, 90 questionnaires have been completed between October 2021 and March 2022. For analysis, descriptive statistics were used and associations between information sources and patients’ lifestyle behaviour explored utilising Spearman’s Rho, Mann–Whitney *U*, and Pearson’s Chi Square tests.

**Results:**

The cancer patients received information on nutrition and PA most frequently from physicians (70.9%), family and friends (68%) and browsing the internet (61.3%). Half of the patients (51.1%) had questions concerning these topics during the time of their disease. The majority of those patients (81.8%) reported that their questions were answered. The topics were addressed primarily with outpatient oncologists (60.0%) and in rehabilitation clinics (53.3%). Just about half of the patients (55.3%) felt satisfactorily informed on nutrition and PA in their cancer disease, more so if they talked to their oncologist or family physician (*Z* = − 2.450, *p* = 0.014 and *Z* = − 3.425, *p* = 0.001 resp.).

**Conclusion:**

Cancer patients receive information on nutrition and PA predominantly after their initial treatment. Since they might be missing significant information to alleviate severe symptoms during their treatment, the importance of nutrition and PA should be emphasised by clinicians early on in treatment.

**Trial registration:**

Trial Registration Number (May 7, 2021): 2021-2149-Bef.

**Supplementary Information:**

The online version contains supplementary material available at 10.1007/s00432-022-04282-w.

## Introduction

A cancer diagnosis sets many cancer patients off on an arduous journey. Managing their disease, treatment, and daily live is a straining endeavour that can leave patients overwhelmed and helpless. Additionally, their information needs are extensive and ever-changing over the course of their treatment (Rutten et al. 2005). Recurring topics during and post-treatment are nutrition and physical activity (PA). In some respects, both lifestyle topics are intertwined with one another. For example, they share similar molecular mechanisms preventing cancer development and progress, contribute to weight gain or loss, and are modifiable by the patients themselves (Wiseman 2019). Furthermore, patients who are physically more active also tend to adhere to a healthier diet, strengthening their association in practice (Grosso et al. 2017; Schlesinger et al. 2014). In cancer patients, nutrition and PA can be negatively affected by both the disease and its treatments. Malnutrition is reported to occur in up to 80% of cancer patients, leading to adverse consequences such as poor survival, functional status, and reduced quality of life (QoL) (Capuano et al. 2010; Datema et al. 2011; Jager-Wittenaar et al. 2011; Lim et al. 2012; Montoya et al. 2010; Mouri et al. 2018; Norman et al. 2010; Pressoir et al. 2010; Wie et al. 2010). Additionally, more than fifty percent of cancer patients report symptoms impacting their nutrition and PA, e.g., early satiety, dysphagia, xerostomia, nausea, vomiting, exercise intolerance, dyspnoea, and fatigue (Deftereos et al. 2021; Maddocks 2020; Trajkovic-Vidakovic et al. 2012). On the other hand, nutrition and PA are modifiable by the patients and in turn allow them to more actively alleviate severe symptoms and side effects and ameliorate their prognosis (Arends et al. 2017; Avancini et al. 2020; Brown et al. 2009, 2012; Bye et al. 2020; Cormie et al. 2017; Edvardsen et al. 2015; Hilfiker et al. 2018; Lee et al. 2016; Maddocks 2020; McTiernan et al. 2019; Montagnese et al. 2020; Mouri et al. 2018; Sarwer et al. 2009; Scott and Tharmalingam 2019; Uster et al. 2018). By providing health information on such topics, patients can be empowered to raise their self-care management skills and engage more actively in shared decision-making (Jung 2014).

In the spirit of patient-centred care, it is desirable to provide patient information that is tailored to their needs to improve healthcare quality (Institute of Medicine (US) Commitee on Quality of Health Care in America 2001). To forward reliable information, it is important to understand where patients receive information from, and whether they are satisfied with the information acquired. Assessing which sources of information cancer patients utilise can help to identify sources with potentially harmful health information. For example, recommendation of one-sided diets as can be found on the internet can lead to malnutrition, harming the patient (Huebner et al. 2014; Suarez-Lledo and Alvarez-Galvez 2021). On the other hand, understanding which sources are preferably utilised by cancer patients, they can be referred specifically to information sources containing reliable information. Previous studies have assessed information needs and sources for cancer patients, but data focussing on information sources with respect to nutrition and PA are lacking (Clarke et al. 2016; Johnston et al. 2021; Lewis et al. 2012; Moldovan-Johnson et al. 2014; Rutten et al. 2005). We aimed at closing this knowledge gap by surveying which information channels on nutrition and PA cancer patients use. Since nutrition and PA are interrelated and often covered in parallel, e.g., in guidelines, booklets, and interventional studies, we examined information sources of both topics simultaneously (Bye et al. 2020; Manneh-Vangramberen 2018; Rock et al. 2022). Furthermore, we wanted to assess with whom they talked about nutrition and PA ever since their cancer diagnosis. Finally, we wanted to assess their self-reported satisfaction regarding their coverage with information.

## Participants and methods

### Questionnaire

We chose to conduct a cross-sectional questionnaire study. A pilot questionnaire was developed in collaboration with physicians, cancer patients, and statisticians. The pilot version was tested for length and comprehension among three cancer patients with different types of cancer. The final questionnaire consisted of 45 questions divided into seven sections in four main categories:

- Demographic data including the cancer diagnosis and time since the diagnosis.
- Assessment of the patient’s lifestyle behaviour (nutrition and PA) and self-efficacy.
- Information sources on nutrition and PA utilised by cancer patients.
- Questions, contact persons (i.e., with whom they talked about nutrition and PA; e.g., physicians, other healthcare practitioners, family and friends), and self-reported satisfaction regarding information on nutrition and PA.

We primarily used closed questions with lists of possible answers in form of Likert scales. Semi-open questions offered the participants space to add and elaborate answers. We utilised the sections *Diet* and *Exercise* of the validated Simple Lifestyle Indicator Questionnaire (SLIQ) by Godwin et al. to assess the patients’ dietary and physical lifestyle behaviour (Godwin et al. 2013). The patients’ self-efficacy was assessed using the validated Short Scale for Measuring General Self-Efficacy Beliefs (ASKU) by Beierlein and colleagues (Beierlein et al. 2012). The CROSS guidelines were utilised to report on the findings (Sharma et al. 2021).

### Patients

Adult German-speaking patients with a cancer diagnosis were eligible to participate. The questionnaire was passed on to a convenience sample of three hospitals (20 questionnaires), four rehabilitation clinics (110 questionnaires), two oncological (40 questionnaires) and two general practices (30 questionnaires) predominantly located in the Federal States of North Rhine-Westphalia and Thuringia, Germany, between October 2021 and March 2022. The participants had the option to complete the survey online, accessible via Soscisurvey over the same period. Participation was voluntarily and anonymous.

### Ethical vote

This study was approved by the Ethics Commission of the University Hospital of Jena (Reg.-Nr. 2021-2149-Bef). All participants gave their consent before enrolling in the study.

### Data preparation and analysis

Out of 200 print versions sent, 90 completed questionnaires were returned (response rate 45%). Additionally, 17 questionnaires have been answered online. Answer forms with more than 25% missing data or missing the type of cancer have been excluded. In total, 90 questionnaires were included in the analysis. Data was collected and analysed using IBM SPSS Statistics 27. During analysis, missing values were excluded listwise.

The analysis focussed on the following main items:

- Information sources: How often do you utilise media (e.g., print media, television, internet-websites) or persons (e.g., physicians, family and friends) to inform yourself on nutrition and physical activity?
- Contact persons: Did you talk to someone about nutrition and physical activity regarding your cancer treatment and follow-up care? If so, who did you talk to? When were these topics addressed? How did you feel when these topics have been addressed?
- Information coverage: How satisfied are you with the information you received which covered nutrition and physical activity during and after your cancer treatment?

We explored whether associations between these items emerged, which could indicate diverse preferences among patient-subgroups. Additionally, we gathered data on demographics, dietary behaviour, physical activity, and patients’ self-efficacy, and assessed associations with either of the main items. To analyse correlations between at least ordinal-scaled items, Spearman’s Rho (effect size *ρ*) was calculated. Mann–Whitney *U* tests were performed to analyse differences in central tendencies between two groups and significances tested asymptotically (*Z:* standardised score, effect size *r* =|*Z|* /√*n*). Associations between nominal variables were analysed utilising Pearson’s Chi Square (*X*^*2*^) tests (*df:* degrees of freedom, Cramér’s *V*: effect size). Effect sizes of correlations and associations were estimated to be small (0.1), medium (0.3) or large (0.5) according to Cohen (Cohen 1988). For all analyses, *p* values smaller than 0.05 were considered significant.

## Results

### Sample

In total, 90 questionnaires have been completed and analysed. Female and male cancer patients participated equally (51.1% or 48.9%), their ages ranging from 18 to 90 years (mean value 58.55 ± 15.74 years, median 60.5 years). The most prevalent types of cancer were leukaemia and lymphomas (23.3%), followed by breast (21.1%) and lung cancer (15.6%). The demographic data are shown in Table 1.

**Table 1:** Selected characteristics of the trial population (*n* = 90)

Criteria	Number	Percentage (%)
Gender		
Female	46	51.1
Male	44	48.9
Age (years)		
Under 40	8	8.9
40–49	12	13.3
50–59	22	24.4
60–69	24	26.7
70–79	12	13.3
Over 80	8	8.9
N.A.	4	4.4
Living in Community with … inhabitants		
Less than 1.000	4	4.4
1.000–9.999	20	22.2
10.000–99.999	26	28.9
100.000-500.000	35	38.9
More than 500.000	3	3.3
N.A.	2	2.2
Marital Status		
Single	7	7.8
Coupled	6	6.7
Married	50	55.6
Divorced	15	16.7
Widowed	11	12.2
N.A.	1	1.1
Attained Education		
Middle School	48	53.3
High School	15	16.7
College/University	27	30.0
Self-estimated Financial Situation		
Below average	13	14.5
Average	55	61.1
Above average	21	23.3
N.A.	1	1.1
Type of Cancer*		
Leukaemia or Lymphoma	21	23.3
Breast Cancer	19	21.1
Lung Cancer	14	15.6
Colon Cancer	9	10.0
Other Gastrointestinal Tumours	9	10.0
Prostate Cancer	6	6.7
Urogenital Carcinomas	6	6.7
Gynaecological Tumours	3	3.3
Other	8	8.9
Time since Cancer Diagnosis		
Less than 3 months	13	14.4
3–6 months	11	12.2
6–12 months	18	20.0
1–3 years	23	25.6
More than 3 years	25	27.8
Respondents received Questionnaire from …		
Rehabilitation Clinic	33	36.7
Oncological Practice	28	31.1
General Practice	9	10.0
Hospital	9	10.0
Other	11	12.2

*Some patients reported more than one type of cancer, therefore exceeding the number of patient cases (*n* = 90). Hence, the percentages *cancer/cases* add up to more than 100%

### Lifestyle behaviour and self-efficacy

**Fig. 1:**
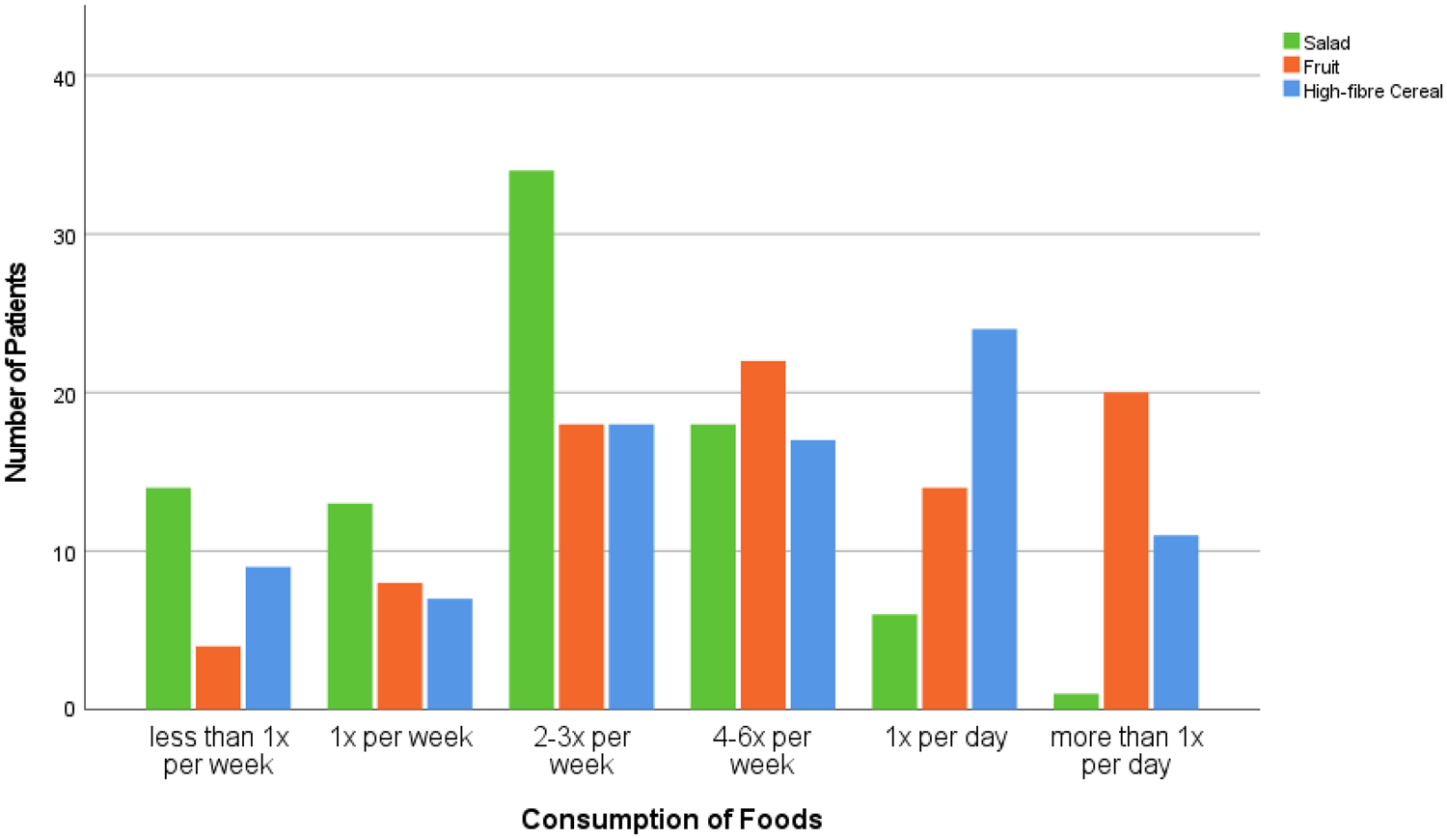
Dietary behaviour of cancer patients as indicated by healthy food-consumption (*n* = 86)

**Fig. 2:**
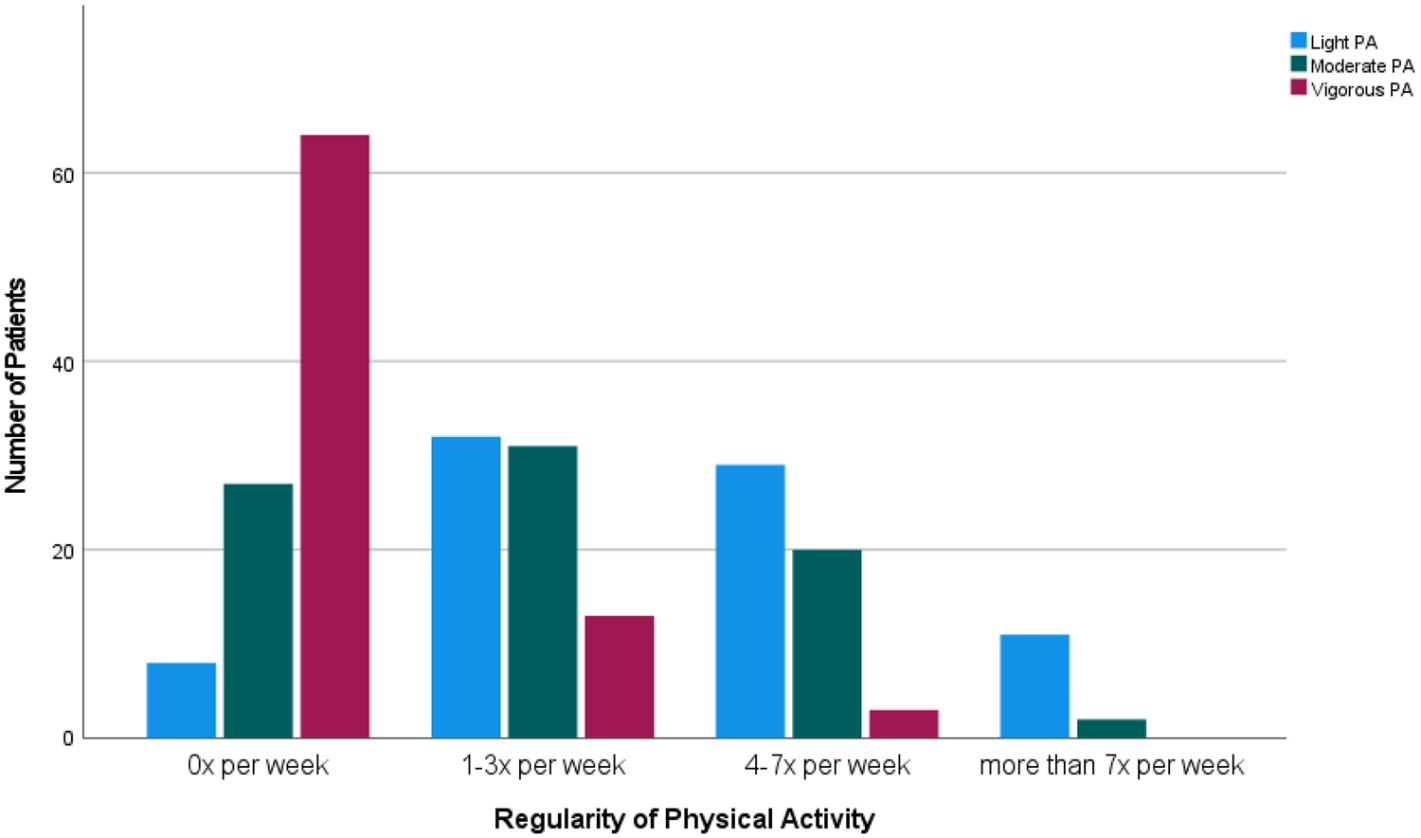
Physical Activity (at least 30 min at a time) of cancer patients (*n* = 80)

The dietary behaviour as reported by the cancer patients is depicted in Fig. 1, while their self-reported PA is subject to Fig. 2. As suggested by the SLIQ, higher consumption of salat, fruit, and high-fibre cereal, as well as higher physical activity indicated a healthier lifestyle (Godwin et al. 2013). Sixty-one percent (61.1%, *n* = 55) of the respondents did not report changes in their dietary behaviour as compared to the time before their cancer disease. Twenty-nine percent (28.9, *n* = 26) consumed aforementioned foods more often, and 10.0% (*n* = 9) less often.

Utilising the ASKU to score cancer patients’ self-efficacy beliefs ranging from 1 (lowest self-efficacy belief) to 5 (highest self-efficacy belief), on average the cancer patients scored 3.84 (± 0.83, median 4, *n* = 87) (Beierlein et al. 2012).

### Information sources on nutrition and physical activity

**Fig. 3:**
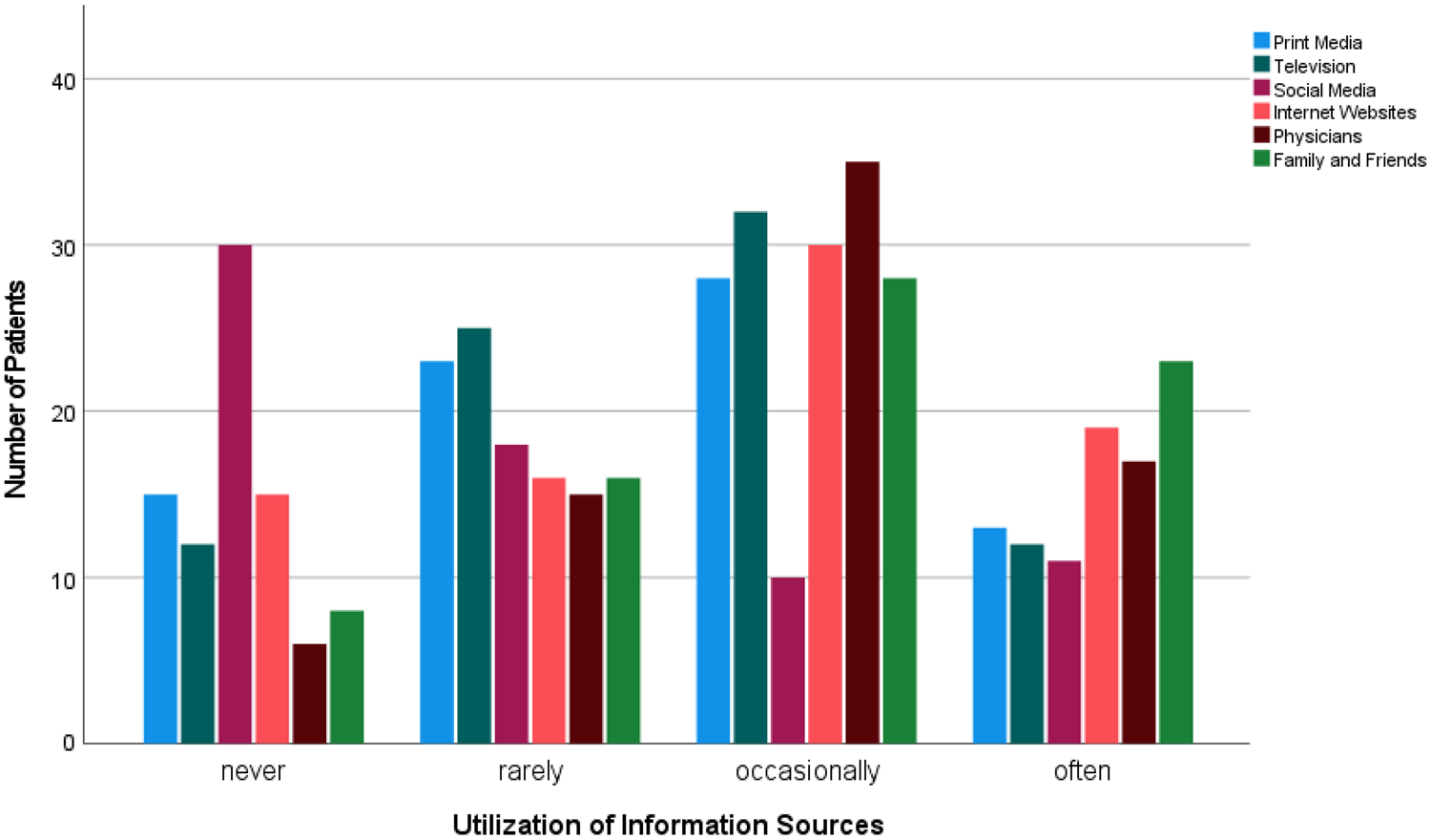
Information sources on nutrition and PA utilised by cancer patients

Cancer patients reported to receive information on nutrition and PA more frequently by physicians, family and friends or through media. Browsing the internet was reported to be the most common medial information source on these topics. Social media, however, was hardly ever utilised. Figure 3 contains the patients’ responses.

Significant correlations between demographics and information sources are presented in Table 2. There was a negative association between the patient’s age and importance of information from internet (*ρ* = − 0.375, *p* = 0.001) and social media (*ρ* = − 0.546, *p* < 0.001). Patients receiving health information through social media also reported a healthier diet (*ρ* = 0.310, *p* = 0.011). The frequency of receiving information from print media was associated with information from television (*ρ* = 0.611, *p* < 0.001). Receiving information from family and friends correlated positively with a healthier lifestyle, i.e., a healthier diet and higher PA (*ρ* = 0.362, *p* = 0.004).

**Table 2:** Significant correlations between demographics and information sources

	Age	Time since diagnosis	SLIQ (healthy lifestyle)	Print media	Television	Social media	Physicians
Print media							
Spearman’s Rho	0.267*	–	–	–	–	–	–
*p* (2-tailed)	0.021	–	–	–	–	–	–
*n*	75	–	–	–	–	–	–
Television							
Spearman’s Rho	–	0.264*	–	0.611**	–	–	–
*p* (2-tailed)	–	0.017	–	0.000	–	–	–
*n*	–	81	–	76	–	–	–
Social media							
Spearman’s Rho	− 0.546**	–	–	–	–	–	–
*p* (2-tailed)	0.000	–	–	–	–	–	–
*n*	66	–	–	–	–	–	–
Internet Websites							
Spearman’s Rho	− 0.375**	–	–	0.306**	–	0.513**	–
*p* (2-tailed)	0.001	–	–	0.008	–	0.000	–
*n*	77	–	–	74	–	67	–
Physicians							
Spearman’s Rho	–	0.291*	–	–	0.254*	–	–
*p* (2-tailed)	–	0.013	–	–	0.033	–	–
*n*	–	73	–	–	71	–	–
Family and friends							
Spearman’s Rho	–	–	0.362**	0.244*	0.325**	–	0.308*
*p* (2-tailed)	–	–	0.004	0.042	0.005	–	0.010
*n*	–	–	63	70	74	–	69

*Correlation is significant at the 0.05 level (2-tailed). **Correlation is significant at the 0.01 level (2-tailed)

### Patient questions and their contact persons

Half of the patients (51.1%, *n* = 45) reported having questions on nutrition and PA with respect to the cancer disease. Most (81.8%, *n* = 36) reported that these questions had been answered, mainly by physicians, dietitians, and sometimes through media like the internet and books.

**Fig. 4:**
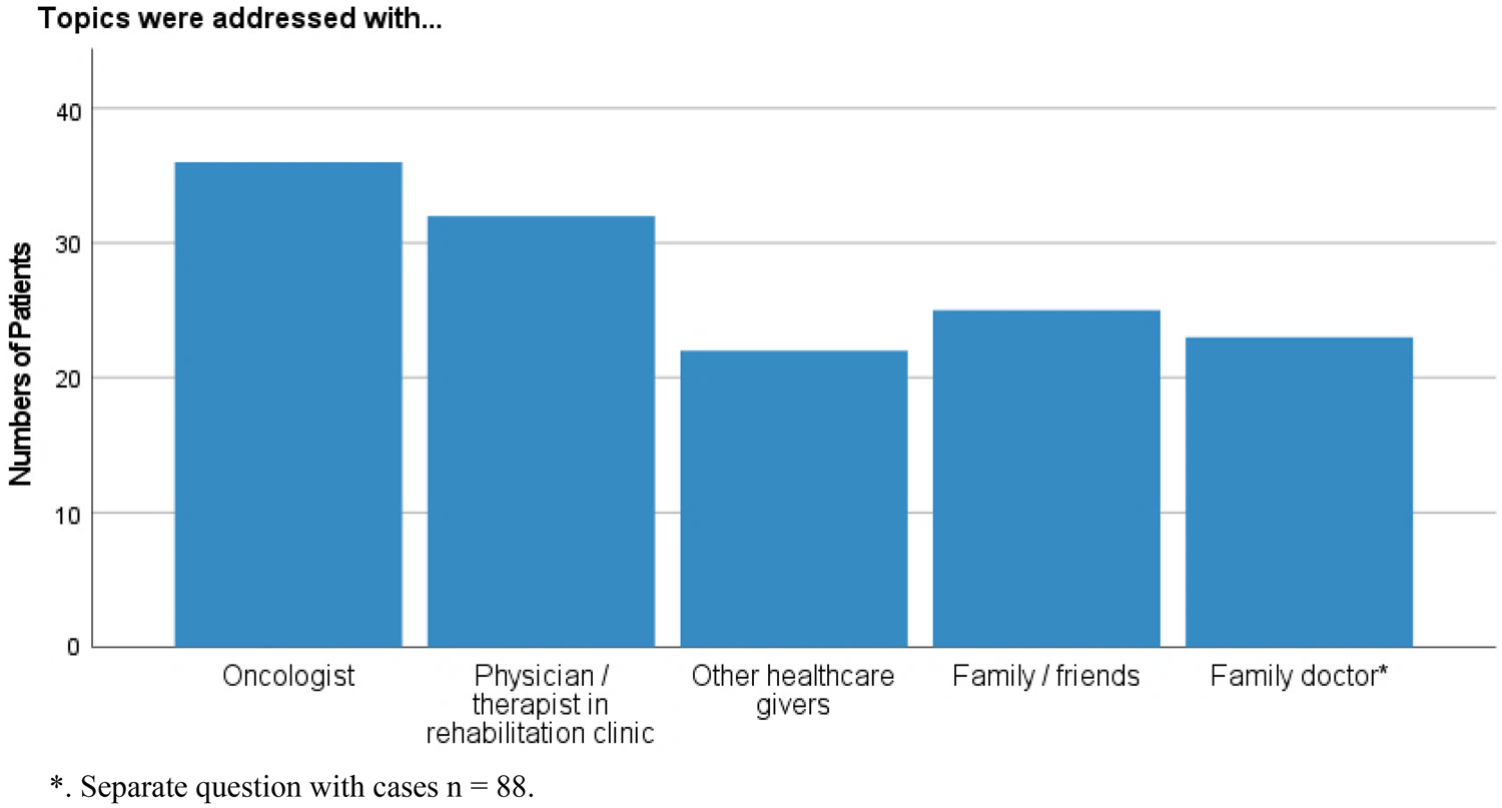
Reference persons of cancer patients concerning nutrition and PA (cases *n* = 60, multiple answers possible). *Separate question with cases *n* = 88

**Fig. 5:**
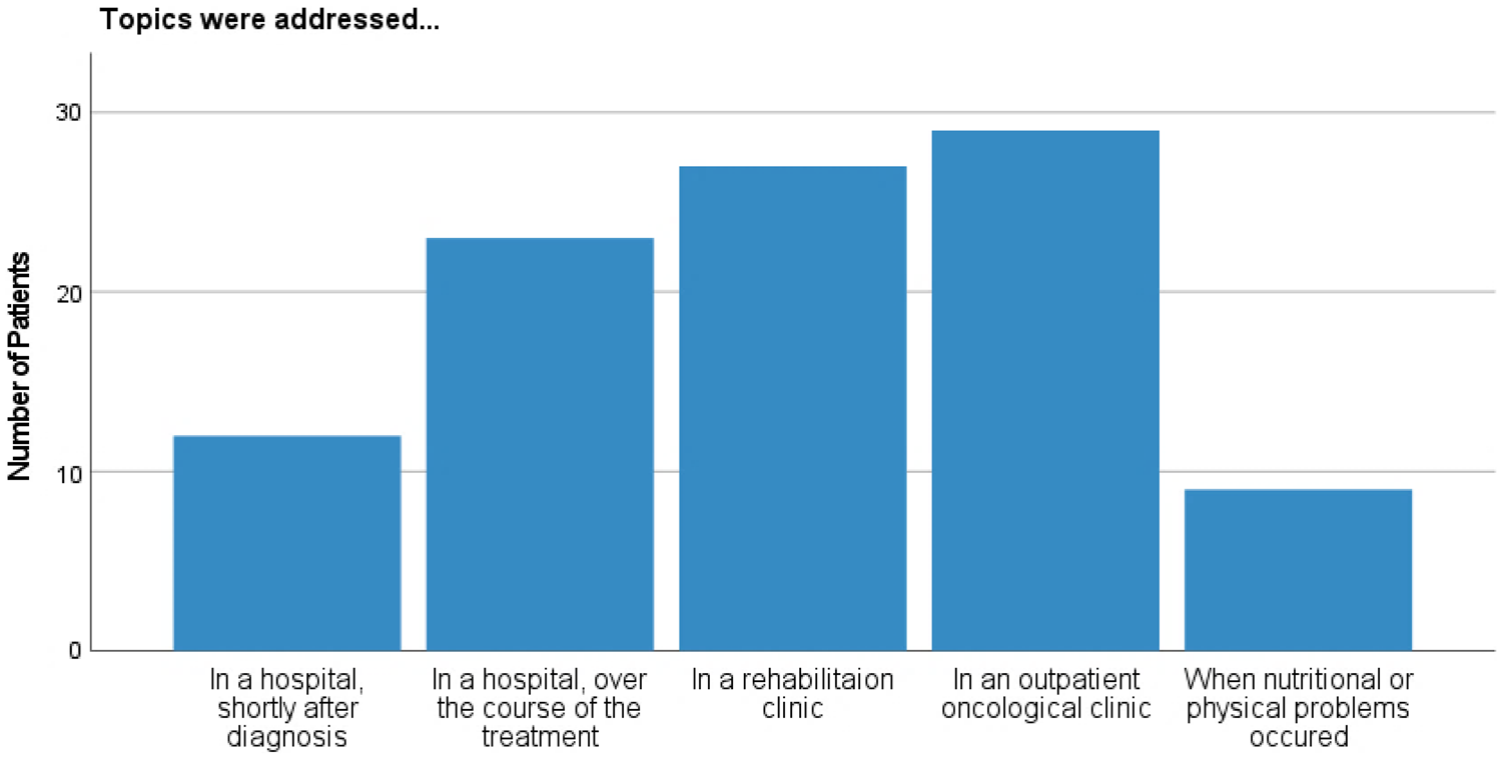
Time and place nutrition and PA have been addressed (cases *n* = 60, multiple answers possible)

Seventy-one percent (71.4%, *n* = 60) talked with someone on nutrition and PA. Their contact persons are listed in Fig. 4. The topics were addressed most frequently in an outpatient clinic by an oncologist or during a stay at a rehabilitation clinic, rather less during a stay in a hospital (see Fig. 5). Twenty percent (20.3%, *n* = 12) felt relieved when nutrition and PA were addressed, whereas 11.9% (*n* = 7) felt rather tense.

A significant association was found between the institution the patient received the questionnaire from, and whom they talked to on nutrition and PA (*X*^*2*^ = 9.930, *df* = 4, 0.042). Men talked significantly more to oncologists than women (*Z* = − 1.996, *r* = 0.258, *p* = 0.046) whereas women and younger patients talked more often with family and friends (*Z* = − 2.740, *r* = 0.354, *p* = 0.006 and *Z* = − 2.306, *r* = 0.303, *p* = 0.021 resp.).

### Self-reported satisfaction with information coverage

More than half of the patients (55.3%, *n* = 47) felt satisfactorily informed on nutrition and PA regarding their cancer disease, whereas 16.5% (*n* = 14) did not feel sufficiently informed.

Patients reported to feel more satisfied with information on nutrition and PA when receiving them from oncologists or family physicians (oncologists: *Z* = − 2.450, *r* = 0.325, *p* = 0.014; family physicians: *Z* = − 3.425, *r* = 0.378, *p* = 0.001). In contrast, no associations occurred for other persons and information sources.

## Discussion

Complementary to studies assessing cancer patients’ information needs and sources, to our knowledge this is the first study focussing on their information sources addressing nutrition and PA since the time of modern communication techniques (Chua et al. 2021; Finney Rutten et al. 2016; Hoh et al. 2018; Rutten et al. 2005; Wieldraaijer et al. 2019). The results indicate that patients commonly have questions concerning nutrition and PA in context of their cancer disease. They usually receive information on these topics from physicians, family and friends, and the internet.

### Information sources

The cancer patients reported that they received information on the topics *nutrition* and *PA* more often by physicians than by media. This corresponds to the findings of Rutten et al. in their systematic review from 2005 regarding information sources of cancer patients (Rutten et al. 2005). Since then, the internet has become more readily available and is utilised more often (Chua et al. 2021). However, healthcare practitioners remain the preferred source of information among cancer patients (Adjei Boakye et al. 2018; Chua et al. 2021; Finney Rutten et al. 2016). In our sample, browsing the internet was the premier medial source of information, and a medium negative correlation with the patient’s age has been found. This association has been observed in other studies as well and could be explained by a higher familiarity of younger patients with the internet (Adjei Boakye et al. 2018; Soto-Perez-de-Celis et al. 2018). A large negative association with the patient’s age emerged concerning social media as source of information, while a large positive correlation between browsing the internet and social media formed. These correlations reflect the fact that younger patients use social media, which is part of the internet, more frequently (Auxier and Anderson 2021). All in all, cancer patients rarely acquired health information via social media channels. Contrary to other studies, no association between information sources and demographic factors other than age were found in our patient sample (Finney Rutten et al. 2016; Kelly et al. 2010; Soto-Perez-de-Celis et al. 2018).

The importance of family and friends as psychosocial support for cancer patients to cope is well established (Lee et al. 2018; Luszczynska et al. 2013; Newton et al. 2021; Rose 1990; Ruiz-Rodríguez et al. 2021). Our study adds an informational role to these contact persons as patients reported to receive information on nutrition and PA from them more often than by media. Interestingly, receiving information from family and friends was correlated positively with a healthier lifestyle regarding nutrition and PA. This finding is in line with data showing higher fruit and vegetable consumption among cancer patients who seek health information from family and friends (Lewis et al. 2012). Support by family and friends furthermore encourages exercise (Lee et al. 2018). They take an active role in the patient’s treatment as they establish the environment in which the patient recovers. Improving lifestyle habits within the patient’s environment would make it easier for the patient to adopt beneficial lifestyle modifications. Thus, family and friends should be included when conveying information to cancer patients.

### Contact persons and information coverage

Half of the cancer patients had questions concerning nutrition and PA regarding their disease, and the majority was answered by healthcare providers. The most common contact persons were oncologists and physicians in rehabilitation clinics, while these topics were rarely brought up visiting their family physician. Information related to rehabilitation such as nutrition and PA are more prevalent post-treatment than during diagnosis and treatment (Rutten et al. 2005). However, they are highly important during cancer therapy as well as many cancer patients experience severe side effects caused by cancer and its treatments (Hébuterne et al. 2014). Many side effects are amendable to PA and nutritional lifestyle modifications, such as cachexia and malnutrition, nausea and vomiting, loss of endurance and loss of power, resulting in improved outcomes and QoL (Arends et al. 2017; Avancini et al. 2020; Brown et al. 2012, 2009; Bye et al. 2020; Cormie et al. 2017; Edvardsen et al. 2015; Hilfiker et al. 2018; Laviano et al. 2018; Lee et al. 2016; Maddocks, 2020; McTiernan et al. 2019; Montagnese et al. 2020; Mouri et al. 2018; Sarwer et al. 2009; Scott and Tharmalingam 2019; Uster et al. 2018). If patients don’t get to know the importance of nutrition and PA, they may be left satisfied because they don’t know what beneficial information they are missing out on. To improve this point, nutrition and PA should become an integral part of cancer diagnosis and treatment. Structured treatment programmes are needed to reliably implement counselling on nutrition and PA in cancer patients and putting advice into practice.

Cancer patients who talked about these topics to their oncologist or family physician expressed significantly higher satisfactions with information received. Whereas they spend time in rehabilitation clinics only temporarily, regular consultations with their oncologist and family physician allow to build trust that is required to improve adherence to a healthy lifestyle and leave the patient satisfied (Baker et al. 2003; Chen et al. 2020; Hendren and Kumagai 2019). While many actors should be part of lifestyle education, one approach is to integrate these physicians in structured treatment programmes to emphasize the opportunities nutrition and PA present.

### Limitations

A higher participation of institutions interested in conveying information on nutrition and PA may have recruited patients who are better informed and more satisfied than the general cancer population. Since most questionnaires were returned from patients in rehabilitation clinics and oncological practices, a recency effect could have biased some items. For example, asking to whom the patients talked to, contact persons other than rehabilitation physicians and oncologists might be underrepresented as they recall their last contact person easier than previous ones.

For the sources of information or reasons for dissatisfaction on these lifestyle topics, we did not differentiate between nutrition and PA, to avoid making the questionnaire too long. We cannot exclude that there are differences between both but have reason to believe that the results are representative since the two topics are interrelated. To provide an overview of information sources utilised by cancer patients, we assessed different types of sources. Since we did not ask for specific sources such as certain websites or books, we cannot assess whether the patients received reliable information. Moreover, we did not ascertain whether the patients put their gathered information into practice.

This study does hardly capture the information sources of patients with advanced cancer, but rather of cancer survivors, resulting in a survivorship bias. Furthermore, we acquired a relatively small number of participants. Therefore, especially sub-items generated very small sample sizes and, at best, produced tendencies. This might compromise generalisability. Finally, socially desired answers cannot be excluded.

Future research should aim to acquire a larger sample size. A longitudinal approach could improve understanding the associations between receiving information from different information sources and changes in cancer patients’ lifestyle behaviour. Information sources should be specified (e.g., which websites cancer patients utilise) and their credibility should be ascertained.

## Conclusion

The transfer of information on nutrition and PA in cancer patients primarily takes place post-treatment, where they receive post-treatment lifestyle advice. This can be problematic as many patients experience nutritional and physical issues already during cancer therapy. Just about half of them are content with the information they received, but they are still potentially missing important information to alleviate severe symptoms by exercise and dietary means. Not receiving this information from healthcare practitioners might entice patients to search for information online, which yields potentially harmful advice. Hence, it is meaningful to forward reliable information as early as possible by credible sources. Clinicians and family physicians should be involved as early in the treatment process as possible. This could be realised as part of structured treatment programmes to emphasize the opportunities nutrition and PA present in cancer treatment. Finally, family and friends should receive the same information to create a favourable environment for the patient to adapt beneficial lifestyle modifications.

## Supplementary Information

Below is the link to the electronic supplementary material.

Supplementary file1 (PDF 222 KB)

## Data Availability

Data available on request from the authors. The data that support the findings of this study are available on request from the corresponding author, S. J. Boesenecker, upon reasonable request.
