# Supplementary material for: Nutrition and physical activity in cancer patients: a survey on their information sources
Source: J Cancer Res Clin Oncol. 2022 Aug 22;149(7):3823–33. doi: 10.1007/s00432-022-04282-w (PMC10314881; doi:10.1007/s00432-022-04282-w)
Supplement: Supplementary file 1 — Supplementary file1 (PDF 222 KB) [file 432_2022_4282_MOESM1_ESM.pdf]

FRIEDRICH-SCHILLER-UNIVERSITÄT JENA

Forschungsprojekt:
Bedeutung des Lebensstils in der Behandlung von Krebspatienten

Liebe Patientin, lieber Patient,

Patienten mit einer Krebsdiagnose haben einen hohen Informationsbedarf. Empfehlungen zur Ernährung und körperlichen Aktivität sind für viele Patienten und Angehörige wichtig. In Zusammenarbeit mit dem Onkologischen Institut am Universitätsklinikum Jena möchte ich mehr über die Kommunikation mit Patienten mit Krebs über diese Themen erfahren. Mithilfe dieser Studie soll die patientengerechte Information über Ernährung und Körperliche Aktivität in der Therapie Krebserkrankter gefördert werden. Dazu möchte ich gerne Ihre Erfahrungen zu diesen Themen erfragen. Es würde mich freuen, Sie für unsere Studie zu gewinnen.

Für die Beantwortung der Fragen brauchen Sie circa 10 Minuten.
Die Teilnahme ist freiwillig. Die Datenerhebung erfolgt anonym.

Sie können den Fragebogen auch im Internet unter der Adresse https://www.soscisurvey.de/lebensstil-2021 oder über den nebenstehenden QR-Code aufrufen.

SCAN MICH

Dem Beiblatt auf der Rückseite können Sie nähere Informationen u.a. zu den Datenschutzbestimmungen sowie dem Ethikvotum entnehmen.

Für die Befragung ist es wichtig, dass möglichst alle Fragen beantwortet werden. Bitte antworten Sie auch dann, wenn Sie nur ein Gefühl oder eine Tendenz zu einer Antwort haben.

Befindet sich hinter einer Antwortmöglichkeit, die Sie angekreuzt haben, ein Pfeil mit einer Nummer (z.B. → weiter bei Frage 4a), springen Sie im Fragebogen zu der entsprechend nummerierten Frage. Dabei übersprungene Fragen brauchen Sie nicht mehr zu beantworten.

Sebastian Bösenecker, Friedrich-Schiller-Universität Jena – 2021
E-Mail: sebastian.boesenecker@uni-jena.de

Beiblatt

Ethikvotum

Die Studie hat das Ethikvotum der Ethikkommission der Friedrich-Schiller-Universität Jena.

Datenschutzbestimmungen

Bei dieser Befragung werden Daten anonymisiert erhoben, gespeichert und ausgewertet. Die Verwendung der Daten erfolgt nach gesetzlichen Bestimmungen und setzt Ihre Zustimmung voraus.

1. Ich erkläre mich einverstanden, dass im Rahmen dieser Befragung anonyme Daten über mich erhoben und in Papierform aufbewahrt sowie auf elektronischen Datenträgern und passwortgeschützten Rechnern zur wissenschaftlichen Auswertung gespeichert werden.
2. Außerdem erkläre ich mich einverstanden, dass autorisierte und zur Verschwiegenheit verpflichtete Personen in die anonymisierten Daten Einsicht nehmen, soweit dies für die Überprüfung der ordnungsgemäßen Durchführung der Studie notwendig ist.
3. Ich bin darüber aufgeklärt, dass ich an der Befragung freiwillig teilnehme und mein Einverständnis erteile. Die Einwilligung zur Erhebung und Verarbeitung meiner anonymen Daten ist unwiderruflich. Aufgrund der anonymen Erhebung der Daten können die gespeicherten Daten rückwirkend nicht mehr gelöscht werden.

Durch das Ausfüllen und die Rückgabe des Fragebogens stimmen Sie der Teilnahme an der Befragung zu.

Verwendete Literatur

Die Frageblöcke 5. und 6. wurden folgender Studie entnommen und adaptiert:
Godwin, M et al. (2008). Testing the Simple Lifestyle Indicator Questionnaire: Initial psychometric study. Can Fam Physician; 54(1):76-7

Der Frageblock 7. wurde aus folgender Studie entnommen:
Beierlein, C; Kovaleva, A et al. (2017). Allgemeine Selbstwirksamkeit Kurzskala (ASUK).

Personenbezeichnungen

Aus Gründen der besseren Lesbarkeit wurde auf die gleichzeitige Verwendung der Sprachformen männlich, weiblich und divers (m/w/d) verzichtet. Sämtliche Personenbezeichnungen gelten gleichermaßen für alle Geschlechter.

1. Zunächst haben wir einige Fragen zu Ihrer Person.

Welches Geschlecht haben Sie?	männlich O	weiblich O	divers O

Wie alt sind Sie? ☐

Wie viele Einwohner hat die Stadt/Gemeinde, in der Sie wohnen?
- O weniger als 1.000
- O 1.000 – 9.999
- O 10.000 – 99.999
- O 100.000 – 500.000
- O mehr als 500.000

Was ist Ihr Familienstand?
- O ledig
- O verheiratet
- O in einer Partnerschaft
- O geschieden
- O verwitwet

Welchen Bildungsabschluss haben Sie?
- O kein Abschluss
- O Haupt-/Realschulabschluss
- O Fach-/Abitur
- O Studium/Fachhochschule

Wie schätzen Sie Ihre finanzielle Situation im Vergleich zur gesamten Bevölkerung ein?

unterdurch-schnittlich		durch-schnittlich		überdurch-schnittlich
O	O	O	O	O

2. Bitte beantworten Sie ein paar Fragen zu Ihrer Krebserkrankung.

Welche Art von Krebs haben Sie? ☐

Wann wurde die Diagnose des Krebses erstmals gestellt?

Vor…
- O weniger als 3 Monaten
- O 3 – 6 Monaten
- O 6 – 12 Monaten
- O 1 – 3 Jahren
- O mehr als 3 Jahren

Welche Therapieformen wurden bislang angewendet?

[Mehrere Antworten sind möglich]
- O Operation
- O Chemotherapie
- O Strahlentherapie
- O antihormonelle Therapie
- O andere Tumormedikamente
- O andere Therapieform: ☐
- O keine davon

3. Im Alltag begegnen uns viele Quellen, die über *Ernährung und Körperliche Aktivität* informieren. Bitte beurteilen Sie, inwieweit Sie aus den folgenden Quellen Informationen beziehen.

Wie häufig beziehen Sie aus folgenden Quellen Informationen über Ernährung und Körperliche Aktivität?	nie	selten	gelegentlich	oft
**Medien (insgesamt)**	O	O	O	O
Davon im Einzelnen:				
Printmedien (z.B. Zeitschriften)	O	O	O	O
Fernsehen	O	O	O	O
Soziale Medien (z.B. YouTube, Facebook)	O	O	O	O
Internet-Websites (z.B. über Google)	O	O	O	O
Öffentliche Bildungsangebote	O	O	O	O
Andere Medien	O	O	O	O
Welche anderen Medien sind das?				

	nie	selten	gelegentlich	oft
**Personen (insgesamt)**	O	O	O	O
Davon im Einzelnen:				
Ärzte	O	O	O	O
Familie und Freunde	O	O	O	O
Andere Personen	O	O	O	O
Welche anderen Personen sind das?				

Haben sich die Informationsquellen seit Ihrer Krebserkrankung verändert?

O Ja
O Nein → **weiter bei Frage 4a**
O Weiß ich nicht → **weiter bei Frage 4a**

Wenn Sie ‚Ja' geantwortet haben:

Folgende Quellen sind neu dazugekommen: ______

Folgende Quellen nutze ich mehr: ______

Folgende Quellen nutze ich seltener: ______

Befindet sich hinter einer Antwortmöglichkeit, die Sie angekreuzt haben, ein Pfeil mit einer Nummer (z.B. → weiter bei 4b), springen Sie im Fragebogen zu der entsprechend nummerierten Frage. Dabei übersprungene Fragen brauchen Sie nicht mehr zu beantworten.

4. Die folgenden Fragen beziehen sich auf die Ernährung und Körperliche Aktivität im Rahmen Ihrer Krebserkrankung.

		unzu-reichend		neutral		aus-reichend
**4a**	Wie umfangreich fühlen Sie sich über Ernährung und Körperliche Aktivität *im Rahmen Ihrer Krebserkrankung* informiert?	O	O	O	O	O
				**→ weiter bei 4b** (neutral bis ausreichend)		

Wenn Sie sich (eher) unzureichend informiert fühlen:

Warum fühlen Sie sich nicht ausreichend informiert?

[Mehrere Antworten sind möglich]

- O Die Informationen waren zu allgemein oder sind nicht auf meine Situation eingegangen.
- O Ich konnte nicht alle Fragen zu den Themen Ernährung und Körperliche Aktivität stellen.
- O Ich habe keinen passenden Ansprechpartner gefunden.
- O Die Informationen, die ich bekommen habe, waren widersprüchlich.
- O Aus einem anderen Grund: ____________________

4b Hatten Sie Fragen zur Ernährung und Körperlichen Aktivität während Ihrer Krebserkrankung?

- O Ja
- O Nein **→ weiter bei 4c**
- O Weiß ich nicht **→ weiter bei 4c**

Wenn Sie ‚Ja' geantwortet haben:

Wurden Ihre Fragen beantwortet?

- O Ja, durch folgende Informationsquellen: ____________________
- O Nein
- O Weiß ich nicht

Weiter auf der Rückseite

**4c**	Wurde mit Ihnen über Ernährung und Körperliche Aktivität *im Rahmen Ihrer Krebserkrankung* gesprochen?	O Ja<br>O Nein<br>O Weiß ich nicht	 → **weiter bei 4d**<br>→ **weiter bei 4d**

Wenn Sie ‚Ja' geantwortet haben:

Mit wem haben Sie über diese Themen gesprochen?<br><br>[Mehrere Antworten sind möglich]	O mit einem Onkologen<br>O mit einem Arzt/Therapeut in einer Reha-Klinik<br>O mit einem Angehörigen eines anderen Gesundheitsberufes<br>O mit einem Familienmitglied/Freund<br>O mit jemand anderem: ________
Wann wurde mit Ihnen über diese Themen gesprochen?<br><br>[Mehrere Antworten sind möglich]	O im Krankenhaus, kurz nach der Diagnosestellung<br>O im Krankenhaus, im Verlauf der Krebstherapie<br>O während eines Reha-Aufenthaltes<br>O ambulant, beim Onkologen<br>O als Probleme mit der Ernährung oder Körperlichen Aktivität aufgetreten sind<br>O zu folgendem Zeitpunkt: ________<br>O zu keiner der genannten Optionen

	erleichtert		neutral		angespannt
Wie haben Sie sich gefühlt, als diese Themen angesprochen wurden?	O	O	O	O	O

**4d**	Sind die Themen Ernährung oder Körperliche Aktivität seit Ihrer Krebsdiagnose im Gespräch mit Ihrem Hausarzt angesprochen worden?	O Ja<br>O Nein<br>O Ich habe keinen Hausarzt<br>O Weiß ich nicht	 → **weiter bei Frage 5**<br>→ **weiter bei Frage 5**<br>→ **weiter bei Frage 5**

Wenn Sie ‚Ja' geantwortet haben:

Wer von Ihnen hat diese Themen **zuerst** angesprochen?	O Ich habe meinen Hausarzt selbst auf Ernährung und Körperliche Aktivität angesprochen.<br>O Mein Hausarzt hat von sich aus Ernährung und Körperliche Aktivität angesprochen.
Hat Ihr Hausarzt auf weitere Ansprechpartner oder Informationsquellen verwiesen?	O Ja<br>O Nein<br>O Weiß ich nicht

5. Um folgende Fragen zu beantworten, betrachten Sie Ihre Essgewohnheiten seit Ihrer Krebserkrankung. Geben Sie an, wie häufig Sie die folgenden Nahrungsmittel verzehrt haben. Bitte beziehen Sie alle Mahl- und Zwischenmahlzeiten sowie auswärts essen gehen (z.B. Restaurant) ein.

**Grüner Salat**<br>(mit oder ohne anderem Gemüse)	O weniger als 1-mal pro Woche<br>O 1-mal pro Woche<br>O 2- bis 3-mal pro Woche<br>O 4- bis 6-mal pro Woche<br>O 1-mal pro Tag<br>O 2-mal oder häufiger pro Tag
**Obst**<br>(inklusive Frisch-, Dosen- oder gefrorenes Obst, aber *keine* Fruchtsäfte)	O weniger als 1-mal pro Woche<br>O 1-mal pro Woche<br>O 2- bis 3-mal pro Woche<br>O 4- bis 6-mal pro Woche<br>O 1-mal pro Tag<br>O 2-mal oder häufiger pro Tag
**Ballaststoffreiches Getreide**<br>(z.B. Haferflocken, Vollkorn-, Roggenbrot)	O weniger als 1-mal pro Woche<br>O 1-mal pro Woche<br>O 2- bis 3-mal pro Woche<br>O 4- bis 6-mal pro Woche<br>O 1-mal pro Tag<br>O 2-mal oder häufiger pro Tag
Haben sich diese Essgewohnheiten **im Vergleich zu vor Ihrer Krebserkrankung** verändert?	O Ja, ich esse oben genannte Nahrungsmittel insgesamt seltener.<br>O Ja, ich esse oben genannte Nahrungsmittel insgesamt häufiger.<br>O Nein, meine Essgewohnheiten haben sich bezüglich oben genannter Nahrungsmittel insgesamt nicht verändert.

Weiter auf der Rückseite

6. Bitte geben Sie an, wie häufig pro Woche Sie seit Ihrer Krebserkrankung folgende Tätigkeiten ausgeführt haben (jeweils für mind. 30 Minuten).

**Leichte Körperliche Aktivität**, darunter bspw.<br>- Leichte Haus- oder Gartenarbeit (Staub wischen, saugen, fegen)<br>- Spazierengehen (z.B. den Hund ausführen)<br>- Bowling, ein Musikinstrument spielen	O 0-mal pro Woche<br>O 1- bis 3-mal pro Woche<br>O 4- bis 7-mal pro Woche<br>O 8-mal oder häufiger pro Woche
**Moderate Körperliche Aktivität**, darunter bspw.<br>- Zügiges Gehen, gemächliches Fahrradfahren, Schwimmen<br>- Moderate Gartenarbeit (Harken, Jäten)<br>- Tanzen, Gymnastikkurse (Tai-Chi, Yoga)	O 0-mal pro Woche<br>O 1- bis 3-mal pro Woche<br>O 4- bis 7-mal pro Woche<br>O 8-mal oder häufiger pro Woche
**Schwere Körperliche Aktivität**, darunter bspw.<br>- Laufen, Joggen, reges Fahrradfahren<br>- Schwere Gartenarbeit, Gewichtheben<br>- Fußball, Basketball u.a. Liga-Sportarten	O 0-mal pro Woche<br>O 1- bis 3-mal pro Woche<br>O 4- bis 7-mal pro Woche<br>O 8-mal oder häufiger pro Woche

7. Die folgenden Aussagen können mehr oder weniger auf Sie zutreffen. Bitte geben Sie bei jeder Aussage an, inwieweit diese auf Sie persönlich zutrifft.

	Trifft gar nicht zu	Trifft wenig zu	Trifft etwas zu	Trifft ziemlich zu	Trifft voll und ganz zu
In schwierigen Situationen kann ich mich auf meine Fähigkeiten verlassen.	O	O	O	O	O
Die meisten Probleme kann ich aus eigener Kraft gut meistern.	O	O	O	O	O
Auch anstrengende und komplizierte Aufgaben kann ich in der Regel gut lösen.	O	O	O	O	O

Herzlichen Dank für Ihre Mithilfe!
